# Outdoor air pollution and terminal duct lobular involution of the normal breast

**DOI:** 10.1186/s13058-020-01339-x

**Published:** 2020-09-24

**Authors:** Nicole M. Niehoff, Alexander P. Keil, Rena R. Jones, Shaoqi Fan, Gretchen L. Gierach, Alexandra J. White

**Affiliations:** 1 Epidemiology Branch, National Institute of Environmental Health Sciences, 111 TW Alexander Drive, Research Triangle Park, NC 27709 USA; 2 Department of Epidemiology, University of North Carolina, Chapel Hill, NC USA; 3 Occupational and Environmental Epidemiology Branch, Division of Cancer Epidemiology and Genetics, National Cancer Institute, Bethesda, MD USA; 4 Integrative Tumor Biology Branch, Division of Cancer Epidemiology and Genetics, National Cancer Institute, Bethesda, MD USA

**Keywords:** Air pollution, Particulate matter, Terminal duct lobular units, Breast involution, Breast cancer

## Abstract

**Background:**

Exposure to certain outdoor air pollutants may be associated with a higher risk of breast cancer, though potential underlying mechanisms are poorly understood. We examined whether outdoor air pollution was associated with involution of terminal duct lobular units (TDLUs), the histologic site where most cancers arise and an intermediate marker of breast cancer risk.

**Methods:**

Pathologist-enumerated TDLUs were assessed in H&E (hematoxylin and eosin)-stained breast tissue sections from 1904 US women ages 18–75 who donated to the Susan G. Komen Tissue Bank (2009–2012). The 2009 annual fine particulate matter < 2.5 μm in diameter (PM_2.5_) total mass (μg/m^3^) at each woman’s residential address was estimated from the Environmental Protection Agency’s Downscaler Model combining Community Multiscale Air Quality (CMAQ) System modeling with air quality monitoring data. We secondarily considered CMAQ-modeled components of PM_2.5_ and gaseous pollutants. We used *K*-means clustering to identify groups of individuals with similar levels of PM_2.5_ components, selecting groups via cluster stability analysis. Relative rates (RRs) and 95% confidence intervals (95% CIs) for the association between air pollutants and TDLU counts were estimated from a zero-inflated negative binomial regression model adjusted for potential confounders.

**Results:**

PM_2.5_ total mass was associated with higher TDLU counts among all women (interquartile range (IQR) increase, RR = 1.06; 95% CI: 1.01–1.11). This association was evident among both premenopausal and postmenopausal women (premenopausal RR = 1.05, 95% CI: 1.00–1.11; postmenopausal RR = 1.11, 95% CI: 1.00–1.23). We identified 3 groups corresponding to clusters that varied geographically and roughly represented high, medium, and low levels of PM_2.5_ components relative to population mean levels. Compared to the cluster with low levels, the clusters with both high (RR = 1.74; 95% CI: 1.08–2.80) and medium (RR = 1.82; 95% CI: 1.13–2.93) levels were associated with higher TDLU counts; although not significantly different, the magnitude of the associations was stronger among postmenopausal women.

**Conclusions:**

Higher PM_2.5_ levels were associated with reduced TDLU involution as measured by TDLU counts. Air pollution exposure may influence the histologic characteristics of normal tissue which could in turn affect breast cancer risk.

## Background

Terminal duct lobular units (TDLUs), the structures within the breast where most cancers arise [1], involute (shrink in number and size) with age. Involution is a complex process that is not observable; however, standardized, reproducible measures to quantify involution have been determined including TDLU count, TDLU span, and number of acini (milk producing substructures) per TDLU. Reduced involution (i.e., higher TDLU count, TDLU span, and acini/TDLU) has been associated with an increased risk of breast cancer among women with benign breast disease [2–4]. Certain established reproductive breast cancer risk factors have been related to TDLU count; later age (≥ 30) at first term birth and lack of breastfeeding have been associated with higher TDLU count in premenopausal women, and earlier age at menarche has been associated with higher TDLU count in postmenopausal women [5]. Circulating sex hormones and estrogen metabolites have also been associated with reduced TDLU involution as measured by TDLU count [6, 7]. Thus, reduced TDLU involution is an intermediate marker of breast cancer risk.

Studies have suggested that outdoor air pollution exposure is related to an increased risk of breast cancer [8]. The most consistent associations have been found for nitrogen dioxide (NO_2_) and nitrogen oxides (NO_x_) [8–12], traffic-derived air pollutants. Although less evidence has been found for fine particulate matter < 2.5 μm in diameter (PM_2.5_) in relation to breast cancer [9, 11, 13, 14], two recent, large epidemiologic studies observed increased risks associated with higher levels [10, 15]. White et al. reported that PM_2.5_ was associated with a higher breast cancer risk overall, for ductal carcinoma in situ, and that there was variability by geographic region and PM_2.5_ component profiles [10]; Villeneuve et al. reported an increased risk of premenopausal breast cancer with higher PM_2.5_ exposure [15]. Additionally, studies have reported associations between multiple different air pollutants (PM_2.5_, airborne metals, polycyclic aromatic hydrocarbons) and increased mammographic density [16, 17], a strong risk factor for breast cancer [18]. Lack of TDLU involution has been associated with higher mammographic density [19–21], and it has been suggested that associations between mammographic density and breast cancer may partially reflect the amount of at-risk epithelium [19, 20]. Therefore, there is support for examining the association between air pollution and TDLU involution. Addressing this question may help inform mechanisms underlying associations between outdoor air pollution and breast cancer risk; however, no epidemiologic studies to date have examined this question.

The objective of this study was to determine whether air pollution was associated with measures of TDLU involution (TDLU count, TDLU span, and acini/TDLU) in normal breast tissue samples from healthy volunteers. We considered PM_2.5_ total mass as the air pollutant of primary interest, but also examined associations for individual components of PM_2.5_, clusters of participants classified by PM_2.5_ component profiles, and gaseous pollutants, including NO_2_ and NO.

## Methods

### Study population

The Komen Tissue Bank (KTB) is a biorepository of breast tissue donated by healthy volunteer women. Additional details have been described elsewhere (https://komentissuebank.iu.edu/). A total of 2,197 women who enrolled in the Komen Tissue Bank between 2009 and 2012 were eligible for the original TDLU cohort [5]. Women were excluded if they did not have available breast tissue (*n* = 10), had a history of cancer (*n* = 177), were pregnant at the time of donation (*n* = 18), did not have information on menopausal status (*n* = 37), or were older than age 75 (*n* = 17). Breast tissue characteristics were measured in the remaining 1,938 tissue samples. Of these, 10 of the samples were from women who donated tissue twice within a year; we only included information from the first tissue donation (*n* = 5), which resulted in 1,933 women with breast tissue characteristics. At the time of donation, women filled out a questionnaire that asked about demographics, medical history, lifestyle factors, and reproductive factors.

All women provided written informed consent, and the protocol was approved by the Indiana University Institutional Review Board.

### Exposure assessment

The primary exposure of interest was the 2009 annual PM_2.5_ level at each woman’s residential address at the time of donation. The Environmental Protection Agency’s Downscaler Model is a Bayesian space-time downscaling fusion model that combines modeled air pollution levels from the Community Multiscale Air Quality (CMAQ) Modeling System with fixed site air pollution measurements from the National Air Monitoring Stations/State and Local Air Monitoring Stations [22]. The 2009 PM_2.5_ concentrations were determined at a 12 × 12 km grid level and linked to each woman’s geocoded residential address. We also considered components of PM_2.5_ (sulfate [SO_4_], nitrate [NO_3_], ammonium [NH_4_], elemental carbon [EC], and organic carbon [OC]) and gaseous pollutants (carbon monoxide [CO], nitrogen oxide [NO], nitrogen dioxide [NO_2_], and sulfur dioxide [SO_2_]). Concentrations of these pollutants were only available from CMAQ modeling (version 5.0.2) [23], but not the more complete Downscaler Model. Therefore, the “raw” (i.e., not combined with air monitoring data) concentrations of PM_2.5_ components and gaseous pollutants were of secondary interest. Although daily measures of these pollutants were also available from the EPA, we considered annual averages because we hypothesized that an annual average would be more biologically relevant for an outcome such as TDLU involution that is likely to reflect cumulative exposure to risk factors over a long latency period. Prior research has shown that former smoking and certain reproductive factors that occurred years prior to breast tissue donation, such as age at menarche and age at first birth, to be associated with TDLU measures [5], supporting the relevance of long-term exposures.

### Outcome assessment

The procedures for the collection of breast tissue and assessment of TDLU measures in the KTB population have been described in detail [5]. Briefly, up to four breast tissue cores were collected from the upper outer quadrant of the breast with a standard 10-gauge needle. One core was fixed in 10% formalin buffer and stained with hematoxylin and eosin (H&E). A pathologist, blinded to other covariates, reviewed the digitized section to enumerate TDLUs (TDLU count) and the percent of fat on the slide (0–25%, 26–50%, 51–75%, 76–100%). Among women with a TDLU count > 0, up to 10 TDLUs were measured for TDLU span (using an electronic ruler (microns)) and acini counts per TDLU in categories (1 = ≤ 10, 2 = 11–20, 3 = 21–30, 4 = 31–50, 5 = > 50). For both TDLU span and acini/TDLU, the median value was calculated across the TDLUs assessed.

Reproducibility of the TDLU assessment was previously evaluated for a subset of 72 randomly selected images and eight duplicate images by three pathologists [5]. Spearman correlations for intra-observer reproducibility of three reviewers were *ρ* = 0.99 for TDLU count, *ρ* = 0.68–0.96 for TDLU span, and *ρ* = 0.79–0.90 for acini/TDLU. Spearman correlations between the reviewers were *ρ* = 0.86–0.96 for TDLU count, *ρ* = 0.66–0.76 for TDLU span, and *ρ* = 0.71–0.76 for acini/TDLU. We considered TDLU count our primary outcome given the higher reproducibility and larger sample size of that measure. However, we included TDLU span and acini/TDLU as secondary outcomes given that the different measures may pertain to different stages of TDLU involution and together provide a more complete picture of the biological processes [5, 7].

### Statistical analyses

To examine the association between each air pollutant and TDLU count, we used zero-inflated negative binomial regression models to estimate relative rates (RRs) and 95% confidence intervals (CIs). The zero-inflated portion of the model was used to account for the fact that 34% of women had a TDLU count of 0, which was higher than what would be expected in standard Poisson and negative-binomial regression models. Negative binomial regression accounted for the larger variance compared to the mean of TDLU count [24]. Median TDLU span (53–266, > 266–382, > 382 units) and acini count/TDLU (1, > 1–2, > 2) were categorized into tertiles. To examine associations between each air pollutant and TDLU span or acini count/TDLU, we used ordinal logistic regression models to estimate odds ratios (ORs) and 95% CIs. We tested the proportional odds assumption with the chi-square score test; violations were not observed. Each air pollutant was examined both continuously per interquartile range (IQR) increase in pollutant concentration and categorically based on quartiles of the pollutant among the study population.

Potential confounders were selected based on the literature and a directed acyclic graph (DAG) [25, 26]; our adjustment set included age (continuous), smoking status (never, former, current), education (high school graduate or less, vocational/technical/associate’s degree, college degree, graduate/professional degree, other), race/ethnicity (non-Hispanic white, non-Hispanic Black, Asian, Hispanic, other), body mass index (< 25.0, 25.0 to < 30.0, ≥ 30.0 kg/m^2^), and percent of fat on the slide (0–25%, 26–50%, 51–75%, 76–100%) (Supplemental Fig. 1). We did not adjust for other TDLU risk factors that are not also related to air pollution exposure, such as number of live births or hormone replacement therapy use, that may affect precision but not control confounding. In the binary, logit portion of the zero-inflated negative binomial models to predict the zero counts, we used the same set of covariates as in the negative binomial portion of the model. This set demonstrated a better model fit than including age alone according to the Akaike information criterion (AIC). Given that conclusions were unchanged regardless of whether the exposures were also included in the zero-inflated portion of the model (data not shown), they were only included in the negative binomial, and not the zero-inflated, portion.

We excluded women whose address was not in the USA or did not provide a donation address (and therefore could not be linked to the air pollution data, *n* = 6) or were missing information on covariates (*n* = 23), leaving 1,904 (98% of those eligible) women for inclusion in our analysis.

We examined associations for TDLU count, TDLU span, and acini/TDLU for all women combined and stratified by menopausal status, using an interaction term between menopausal status and the air pollutant. Women were classified as postmenopausal (*n* = 574) if they reported that they had not had a menstrual period in the past 12 months, had a bilateral oophorectomy, had a hysterectomy without a bilateral oophorectomy and were ≥ 55 years old, or had a uterine ablation and were ≥ 55 years old. Women were classified as premenopausal (*n* = 1,322) if they reported having a period in the past 12 months, no history of bilateral oophorectomy, history of a hysterectomy without a bilateral oophorectomy and were < 55 years old, or a uterine ablation and were < 55 years old. Eight women were missing information on menopausal status; these women were included in the analyses for the overall population but were not included in the analyses stratified on menopausal status.

Given that a majority (89%) of the women in this population lived in Indiana at the time of tissue donation and that outdoor air pollution sources may vary between Indiana and the rest of the USA, we conducted a sensitivity analysis restricted to Indiana residents (TDLU count only). Individual pollutant analyses were conducted in SAS 9.4 (Cary, NC).

We used *K*-means clustering to identify groups of individuals with similar patterns of PM_2.5_ component exposure. *K*-means is an unsupervised clustering method to identify a pre-specified number (*k*) of representative joint exposure values (centroids) that can be used to classify individuals into *k* groups according to which centroid is closest to an individual’s exposure [27]. We examined a plot of the proportion of within over total sums of squares by number of clusters and used cluster stability analysis to determine the smallest number of clusters with the highest Rand index [28]. Roughly, the Rand index selects the value of *k* that maximizes the repeatability of clustering across subsets of the population and is seen as a measure of how robust the clustering algorithm is to variability in the population. The number of clusters identified at the bend in the plot of the within over the total sums of squares was five, but the Rand index identified three clusters as optimal. Given that the difference in the proportion of the within over total sums of squares between three and five clusters was small, we used three clusters. Identification of the clusters was completed in R 3.6.0.

We then described the clusters by comparing the mean concentrations of the PM_2.5_ components across the individuals in each cluster to the population means. We compared participant characteristics and geographic variation across the clusters. We examined the association between the clusters and TDLU count using the same analytic methods as described above. One of the clusters had a smaller number of women and given that the TDLU span and acini/TDLU analyses were only among those with a TDLU count > 0, we did not have sufficient power to examine the association between cluster and TDLU span and acini/TDLU.

## Results

### Population characteristics

The average age of the 1,904 women included in this study was 41.5 years (Table 1). Overall, 72% of the women were non-Hispanic white, 58% had a college degree or higher, 73% had never smoked, and 37% had a BMI ≥ 30.0 kg/m^2^. Compared to postmenopausal women, women who were premenopausal at tissue donation were more likely to have a college degree or higher, be never smokers, have a lower percent of fat on the slide, and have a BMI < 25.0 kg/m^2^. The state of residence of the women at the time of their tissue donation is provided in Supplemental Table 1.

**Table 1:** Characteristics of the 1,904 US-based women with TDLU information from the Komen Tissue Bank

	Overall (***n*** = 1,904)^a^		Premenopausal (***n*** = 1,322)		Postmenopausal (***n*** = 574)	
	***N***	%	***N***	%	***N***	%
**Age (years) at donation (mean, SD)**	41.5	13.8	34.9	10.0	56.9	8.2
**Race/ethnicity**						
Non-Hispanic white	1,370	72	946	72	420	73
Non-Hispanic Black	355	19	235	18	120	21
Asian	29	2	22	2	7	1
Hispanic	134	7	107	8	23	4
Other	16	1	12	1	4	1
**Highest level of education**						
Less than high school or high school graduate	383	20	278	21	103	18
Vocational or technical school or associate’s	288	15	175	13	110	19
College degree	653	34	503	38	147	26
Graduate or professional degree	456	24	289	22	167	29
Other	124	7	77	6	47	8
**Cigarette smoking status**						
Never	1,388	73	1,012	76	370	65
Past	388	20	216	16	170	30
Current	128	7	94	7	34	6
**Percent of fat on the slide**						
0–25%	170	9	150	11	18	3
26–50%	178	9	142	11	35	6
51–75%	366	19	281	21	84	15
76–100%	1,190	63	749	57	437	76
**BMI**						
< 25.0	666	35	521	39	144	25
25.0 to < 30.0	530	28	338	26	189	33
≥ 30.0	708	37	463	35	241	42
**Year of tissue donation**						
2009	322	16	247	19	69	12
2010	331	17	236	18	93	16
2011	451	24	315	24	136	24
2012	800	42	524	40	276	48
**TDLU count (mean, SD)**	7.3	10.3	8.4	11.1	4.5	7.3
**TDLU span (mean, SD)**^b^	349.7	164.5	374.1	165.9	280.2	137.7
**Category of acini/TDLU (mean, SD)**^b^	1.6	0.8	1.8	0.9	1.3	0.6

Percentages may not sum to 100% due to rounding

^a^Eight were missing menopausal status, but were included in the overall population

^b^Among the 1,262 women (926 premenopausal, 330 postmenopausal) with a TDLU count > 0

### Air pollutant characteristics

The 2009 annual mean residential PM_2.5_ total mass was 12.5 μg/m^3^ (standard deviation (SD) = 0.7 μg/m^3^) (Additional file 1, Supplemental Table 2). SO_4_ was the PM_2.5_ component with the highest concentration (mean = 2.4 μg/m^3^); CO was the gaseous pollutant with the highest concentration (mean = 223.4 ppb). The strongest correlations among the pollutants examined were between EC, NO, and NO_2_ (*r* = 0.99 and 0.98), and the weakest correlation was between NO_3_ and SO_4_ (*r* = 0.02) (Additional file 1, Supplemental Fig. 2).

### PM_2.5_ total mass and TDLU involution measures

An IQR increase in PM_2.5_ total mass (0.5 μg/m^3^) was associated with higher TDLU count among all women (RR = 1.06; 95% CI: 1.01, 1.11) (Table 2). This association was evident in both premenopausal (RR = 1.05; 95% CI: 1.00, 1.11) and postmenopausal (RR = 1.11; 95% CI: 1.00, 1.23) women. Quartiles of PM_2.5_ above the referent were associated with an elevated TDLU count among all women, although the association was non-linear with stronger associations in quartile 2 and quartile 3 than in quartile 4 (e.g., quartile 2 vs. 1 RR = 1.17; 95% CI: 1.00, 1.38). The associations between PM_2.5_ total mass and TDLU count were similar when restricted to Indiana residents among all women (Additional file 1, Supplemental Table 3). In premenopausal women, the quartile-based associations were slightly stronger whereas among postmenopausal women the associations were slightly attenuated among Indiana residents. However, there was considerable overlap in the CIs between the estimates for the overall population and the Indiana residents. PM_2.5_ total mass was not associated with TDLU span (Additional file 1, Supplemental Table 4). A positive, non-statistically significant association with acini count/TDLU was observed for an IQR increase in PM_2.5_ total mass (OR = 1.06; 95% CI: 0.96, 1.17), but this association was not observed for PM_2.5_ categorized in quartiles and did not significantly vary by menopausal status (Additional file 1, Supplemental Table 5).

**Table 2:** Associations between PM_2.5_ total mass and terminal duct lobular unit (TDLU) counts

	All women		Premenopausal		Postmenopausal	
	***N***	RR^a,b^ (95% CI)	***N***	RR^a,b^ (95% CI)	***N***	RR^a,b^ (95% CI)
**PM**_**2.5**_ **total mass (μg/m**^**3**^**)**						
Quartile 1	477	Ref.	333	Ref.	143	Ref.
Quartile 2	464	1.17 (1.00, 1.38)	333	1.21 (1.00, 1.45)	126	1.02 (0.72, 1.42)
Quartile 3	529	1.16 (0.99, 1.37)	337	1.21 (1.00, 1.47)	190	1.06 (0.78, 1.44)
Quartile 4	434	1.08 (0.92, 1.28)	319	1.04 (0.86, 1.26)	115	1.28 (0.92, 1.78)
IQR increase^c^	1,904	1.06 (1.01, 1.11)	1,322	1.05 (1.00, 1.11)	574	1.11 (1.00, 1.23)

PM_2.5_ total mass concentrations (μg/m^3^) were estimated by the EPA’s Downscaler Model of fused CMAQ and monitoring data

^a^Adjusted for age, smoking status, education, race/ethnicity, BMI, and percent of fat on the slide

^b^From a zero-inflated negative binomial regression model. Zero model based on same covariates as main model

^c^IQR increase = 0.5 μg/m^3^

### PM_2.5_ components and TDLU involution measures

An IQR increase in all components of PM_2.5_ (SO_4_, NO_3_, NH_4_, EC), except for OC, was associated with higher TDLU count among all women, although these associations were not statistically significant (Table 3). The 4^th^ vs. 1^st^ quartile of NO_3_ (RR = 1.28; 95% CI: 0.92, 1.77) and NH_4_ (RR = 1.39; 95% CI: 1.01, 1.91) were associated with higher TDLU counts only among postmenopausal women, although there was no statistically significant heterogeneity in associations by menopausal status. The associations were slightly attenuated in the sensitivity analysis restricted to Indiana residents, with the exception of stronger associations for the 2^nd^ and 3^rd^ quartile of EC and OC overall and in premenopausal women (Additional file 1, Supplemental Table 6).

**Table 3:** Associations between PM_2.5_ components and gaseous pollutants and terminal duct lobular unit (TDLU) counts

	All women		Premenopausal		Postmenopausal	
	***N***	RR^a,b^ (95% CI)	***N***	RR^a,b^ (95% CI)	***N***	RR^a,b^ (95% CI)
**PM**_**2.5**_ **components (μg/m**^**3**^**)**						
**SO**_**4**_						
Quartile 1	446	Ref.	323	Ref.	121	Ref.
Quartile 2	526	1.20 (1.02, 1.40)	367	1.23 (1.02, 1.47)	158	1.13 (0.82, 1.54)
Quartile 3	465	0.98 (0.84, 1.15)	303	0.99 (0.82, 1.20)	160	0.99 (0.72, 1.36)
Quartile 4	467	1.10 (0.93, 1.29)	329	1.14 (0.94, 1.37)	135	1.01 (0.72, 1.44)
IQR increase^c^	1,904	1.04 (0.99, 1.08)	1,322	1.04 (0.99, 1.08)	574	1.05 (0.96, 1.14)
**NO**_**3**_						
Quartile 1	476	Ref.	319	Ref.	153	Ref.
Quartile 2	444	1.00 (0.85, 1.17)	318	0.95 (0.79, 1.14)	122	1.19 (0.85, 1.68)
Quartile 3	512	1.05 (0.90, 1.23)	354	1.00 (0.83, 1.20)	155	1.26 (0.91, 1.74)
Quartile 4	476	1.08 (0.92, 1.27)	331	1.04 (0.86, 1.25)	144	1.28 (0.92, 1.77)
IQR increase^c^	1,904	1.03 (0.98, 1.09)	1,322	1.01 (0.96, 1.08)	574	1.10 (0.99, 1.23)
**NH**_**4**_						
Quartile 1	478	Ref.	311	Ref.	163	Ref.
Quartile 2	467	1.08 (0.92, 1.27)	345	1.02 (0.84, 1.22)	121	1.29 (0.92, 1.79)
Quartile 3	551	0.97 (0.82, 1.14)	293	0.94 (0.78, 1.14)	155	1.09 (0.79, 1.50)
Quartile 4	508	1.07 (0.91, 1.25)	373	1.00 (0.83, 1.20)	135	1.39 (1.01, 1.91)
IQR increase^c^	1,904	1.05 (0.99, 1.11)	1,322	1.03 (0.96, 1.10)	574	1.15 (1.02, 1.30)
**EC**						
Quartile 1	474	Ref.	348	Ref.	125	Ref.
Quartile 2	507	1.23 (1.05, 1.44)	359	1.27 (1.07, 1.52)	143	1.08 (0.77, 1.50)
Quartile 3	406	1.02 (0.85, 1.21)	260	1.03 (0.84, 1.26)	144	1.01 (0.72, 1.40)
Quartile 4	517	1.11 (0.94, 1.30)	355	1.10 (0.92, 1.32)	162	1.17 (0.85, 1.61)
IQR increase^c^	1,904	1.05 (0.95, 1.15)	1,322	1.04 (0.94, 1.16)	574	1.09 (0.91, 1.31)
**OC**						
Quartile 1	477	Ref.	341	Ref.	134	Ref.
Quartile 2	499	1.11 (0.95, 1.30)	361	1.13 (0.95, 1.36)	136	1.07 (0.77, 1.48)
Quartile 3	424	1.12 (0.95, 1.32)	272	1.18 (0.97, 1.43)	150	0.99 (0.71, 1.36)
Quartile 4	504	1.02 (0.87, 1.20)	348	1.03 (0.86, 1.24)	154	1.03 (0.75, 1.41)
IQR increase^c^	1,904	1.01 (0.91, 1.11)	1,322	1.01 (0.90, 1.13)	574	1.05 (0.85, 1.29)
**Gaseous pollutants (ppb)**						
**CO**						
Quartile 1	474	Ref.	342	Ref.	131	Ref.
Quartile 2	510	1.18 (1.01, 1.39)	354	1.24 (1.03, 1.48)	152	1.00 (0.72, 1.39)
Quartile 3	459	1.09 (0.93, 1.29)	317	1.15 (0.95, 1.38)	141	0.94 (0.68, 1.31)
Quartile 4	561	1.08 (0.91, 1.27)	309	1.06 (0.88, 1.29)	150	1.11 (0.81, 1.52)
IQR increase^c^	1,904	1.01 (0.92, 1.11)	1,322	1.01 (0.90, 1.12)	574	1.06 (0.87, 1.28)
**NO**						
Quartile 1	479	Ref.	350	Ref.	126	Ref.
Quartile 2	471	1.21 (1.03, 1.41)	336	1.22 (1.02, 1.46)	132	1.12 (0.79, 1.57)
Quartile 3	428	1.03 (0.87, 1.22)	274	1.06 (0.87, 1.29)	152	1.00 (0.73, 1.37)
Quartile 4	526	1.08 (0.92, 1.27)	362	1.07 (0.89, 1.28)	164	1.17 (0.85, 1.60)
IQR increase^c^	1,904	1.02 (0.94, 1.12)	1,322	1.02 (0.92, 1.12)	574	1.08 (0.91, 1.29)
**NO**_**2**_						
Quartile 1	474	Ref.	344	Ref.	129	Ref.
Quartile 2	480	1.23 (1.05, 1.44)	339	1.28 (1.07, 1.54)	137	1.02 (0.73, 1.43)
Quartile 3	491	1.05 (0.89, 1.24)	327	1.08 (0.89, 1.30)	161	1.00 (0.73, 1.37)
Quartile 4	459	1.12 (0.95, 1.33)	312	1.13 (0.94, 1.37)	147	1.11 (0.80, 1.54)
IQR increase^c^	1,904	1.03 (0.95, 1.13)	1,322	1.03 (0.93, 1.14)	574	1.07 (0.90, 1.26)
**SO**_**2**_						
Quartile 1	470	Ref.	323	Ref.	146	Ref.
Quartile 2	435	1.20 (1.02, 1.41)	309	1.24 (1.02, 1.49)	124	1.02 (0.73, 1.42)
Quartile 3	528	1.05 (0.90, 1.22)	355	1.02 (0.85, 1.22)	171	1.14 (0.84, 1.54)
Quartile 4	471	1.04 (0.89, 1.23)	335	1.07 (0.88, 1.29)	133	0.97 (0.71, 1.34)
IQR increase^c^	1,904	1.01 (0.94, 1.07)	1,322	1.00 (0.93, 1.08)	574	1.05 (0.92, 1.20)

PM_2.5_ component (μg/m^3^) and gaseous pollutant concentrations (ppb) were estimated from “raw” CMAQ data

^a^Adjusted for age, smoking status, education, race/ethnicity, BMI, and percent of fat on the slide

^b^From a zero-inflated negative binomial regression model. Zero model based on same covariates as main model

^c^IQR increase: SO_4_ = 0.1 μg/m^3^, NO_3_ = 0.3 μg/m^3^, NH_4_ = 0.1 μg/m^3^, EC = 0.8 μg/m^3^, OC = 0.8 μg/m^3^, CO = 85.6 ppb, NO = 5.0 ppb, NO_2_ = 7.1 ppb, SO_2_ = 0.8 ppb

A 4^th^ vs. 1^st^ quartile increase in NO_3_ (OR = 0.71; 95% CI: 0.52, 0.97), NH_4_ (OR = 0.63; 95% CI: 0.47, 0.86), and EC (OR = 0.73; 95% CI: 0.54, 0.99) and an IQR increase in EC (OR = 0.80; 95% CI: 0.67, 0.96) and OC (OR = 0.81; 95% CI: 0.67, 0.97) were inversely associated with TDLU span among all women (Additional file 1, Supplemental Table 7). PM_2.5_ components were not associated with acini count/TDLU (Additional file 1, Supplemental Table 8).

### Gaseous pollutants and TDLU involution measures

All gaseous pollutants associated with higher TDLU count in a non-linear manner, with significant associations in the 2^nd^ vs. 1^st^ quartile for all women and premenopausal women (Table 3). Except for SO_2_, the associations were slightly stronger when restricted to Indiana residents (Additional file 1, Supplemental Table 6).

IQR increases in gaseous pollutants, except for SO_4_, were inversely associated with TDLU span, but not acini count/TDLU (Additional file 1, Supplemental Tables 7 and 8).

### PM_2.5_ component clusters and TDLU involution measures

**Fig. 1:**
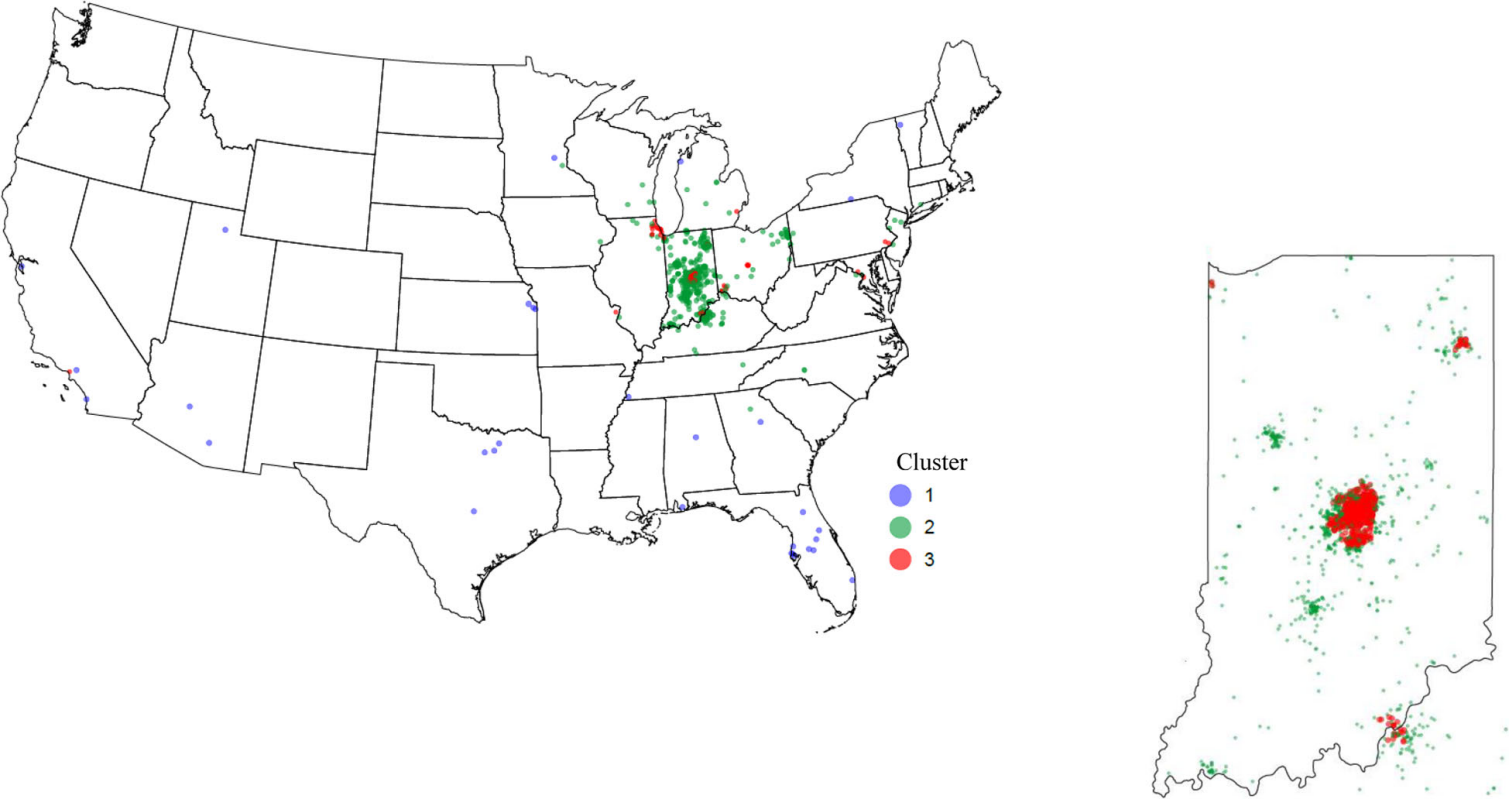
Maps of the residential addresses of Komen women at the time of tissue donation according to PM_2.5_ component cluster

Using *K*-means, three clusters were identified that roughly represented high, medium, and low levels of all PM_2.5_ components. These clusters followed distinct geographic patterns (Fig. 1, Supplemental Table 9) and followed patterns of demographic characteristics. Cluster 1 was composed of 31 individuals who only lived in states other than Indiana; cluster 2 included 1,058 individuals who mostly lived in other parts of Indiana besides Indianapolis and also in a few other states, particularly Ohio and Kentucky; and cluster 3 consisted of 815 individuals who were mostly concentrated around Indianapolis, IN, and around urban areas of surrounding states such as Louisville, KY; Cincinnati and Columbus, OH; and Chicago, IL (Fig. 1). Average levels of PM_2.5_ components among cluster 1 individuals were lower than levels in the study population as a whole (Additional file 1, Supplemental Table 10). These individuals were more likely to have a graduate or professional degree, be past smokers, and have a BMI < 25.0 kg/m^2^ compared to the other clusters. Levels of PM_2.5_ components among cluster 2 individuals were similar to the overall population for SO_4_, NO_3,_ and NH_4_, but slightly lower for EC and OC. Individuals in this cluster were more likely to be non-Hispanic white and have a high school degree or less. Levels of all PM_2.5_ components among cluster 3 individuals were higher than the population mean levels, and this group was more likely to be non-Hispanic Black.

Compared to cluster 1, which had lower concentrations of PM_2.5_ components, clusters 2 (RR = 1.82; 95% CI: 1.13, 2.93) and 3 (RR = 1.74; 95% CI: 1.08, 2.80) were associated with higher TDLU count among all women (Table 4). The magnitude of these associations was stronger among postmenopausal (cluster 3 vs. 1 RR = 3.33; 95% CI: 1.19, 9.35) compared to premenopausal women (cluster 3 vs. 1 RR = 1.56; 95% CI: 0.91, 2.68), although there was no statistically significant heterogeneity by menopausal status.

**Table 4:** Associations between PM_2.5_ component clusters and TDLU count

	All women		Premenopausal		Postmenopausal	
	***N***	RR^a,b^ (95% CI)	***N***	RR^a,b^ (95% CI)	***N***	RR^a,b^ (95% CI)
**Cluster**						
1	31	Ref.	18	Ref.	13	Ref.
2	1058	1.82 (1.13, 2.93)	750	1.64 (0.96, 2.80)	302	3.30 (1.17, 9.28)
3	815	1.74 (1.08, 2.80)	554	1.56 (0.91, 2.68)	259	3.33 (1.19, 9.35)

^a^Adjusted for age, smoking status, education, race/ethnicity, BMI, and percent of fat on the slide

^b^From a zero-inflated negative binomial regression model. Zero model based on same covariates as main model

## Discussion

In this study of women who volunteered to donate healthy breast tissue to the Komen Tissue Bank, we found that living in areas of higher exposure to PM_2.5_ was associated with reduced involution of the breast as measured by higher TDLU count. Consistent with this, most of the assessed individual components of PM_2.5_ were non-linearly associated with higher TDLU count and clusters of individuals with levels of all PM_2.5_ components above or at study population mean levels had higher TDLU counts compared to the cluster with levels below the population mean. Reduced TDLU involution has been associated with an increased risk of breast cancer and may reflect higher amounts of at-risk epithelium. Therefore, our results suggest PM_2.5_ could impact the histologic characteristics of breast tissue and inform early carcinogenic mechanisms relating air pollution to breast cancer risk.

To our knowledge, this was the first study to examine the association between air pollution and TDLU involution measures. While there is little prior epidemiologic work with which to compare our results, biologic and indirectly related results support our overall finding that PM_2.5_ is associated with reduced involution of the breast. Reproductive factors, estrogen metabolites, and circulating sex hormones have all been associated with TDLU count, which suggests that involution of the breast may partially occur through a hormone-related pathway [5–7]. Estrogenic and antiestrogenic effects of airborne particles were reported in a study of human T47D-KBluc breast cancer cells [29], particulate matter < 1 μm in diameter is composed of compounds that affect estrogen-regulated pathways in vivo [30], and genotoxic effects of PM_2.5_ have been reported in mice [31]. Polycyclic aromatic hydrocarbons and metals, components of PM_2.5_ that we were unable to evaluate here but may have contributed to our PM_2.5_ total mass finding, have been shown to be estrogenic and induce mammary tumors in animal models [32, 33]. Additionally, among breast cancer patients, higher levels of multiple pro-inflammatory markers were associated with reduced involution of healthy breast tissue [34]. PM_2.5_ has been shown to increase systemic inflammation, as measured by C-reactive protein [35, 36].

Although earlier epidemiologic studies of PM_2.5_ and breast cancer did not report evidence of elevated risk [9, 11, 13, 14], two recent large cohort studies found PM_2.5_ was associated with an increased risk of breast cancer [15], and that composition of PM_2.5_ and geographic variability were important [10]. Further, in a Breast Cancer Surveillance Consortium study of 279,967 women, PM_2.5_ was positively associated with mammographic breast density (i.e., heterogeneously dense compared to scattered fibroglandular breasts) [16], although a smaller study reported no significant association [37]. Mammographic density is one of the strongest known breast cancer risk factors [18], and reduced TDLU involution has been associated with higher breast density [19, 21]. One hypothesis for the relationship between mammographic density and breast cancer risk is that it may be due in part to the amount of at-risk epithelium such as that measured by TDLU involution [19]. Therefore, our results suggest exposure to PM_2.5_ may impact characteristics of healthy breast tissue, which could influence future breast cancer risk.

In addition to PM_2.5_ and its components, we found that the gaseous pollutants, CO, NO, NO_2_, and SO_2_, were non-linearly associated with elevated TDLU count. CO, NO, and NO_2_ are common traffic-related air pollutants, and NO and NO_2_ are the pollutants that have been most consistently associated with an increased risk of breast cancer [8]. While biological mechanisms linking NO and NO_2_ to TDLU involution are not established, these pollutants may be proxies for other traffic-related air pollutants such as polycyclic aromatic hydrocarbons, which have demonstrated both antiestrogenic and estrogenic activity [38].

PM_2.5_ total mass was not consistently associated with TDLU span or acini count/TDLU. However, individual components of PM_2.5_, except for SO_4_, and gaseous pollutants were inversely associated with TDLU span but not associated with acini count/TDLU. Differences in the associations across measures may be because TDLU span and acini count/TDLU were only assessed among women who had > 0 TDLUs, so there was reduced statistical power for these analyses. The underlying biologic mechanisms and significance of the inverse associations generally observed in relation to TDLU span, in contrast to the positive associations in relation to TDLU count, are unclear. However, as suggested previously, there is a weak correlation between TDLU count and acini count/TDLU or TDLU span and associations for certain other factors also vary by TDLU measure [5]. For example, parity and higher levels of circulating sex hormones were associated with TDLU counts but not TDLU span or acini/TDLU [5, 6]. Compared to never use of hormone replacement therapy in postmenopausal women, current use was associated with TDLU span but not TDLU count or acini/TDLU while former use was associated with TDLU count and span but not acini/TDLU [5]. Therefore, the different markers of involution may represent distinct processes or stages of involution (i.e., complete disappearance vs reduction in size) [5, 7, 39] and more research is needed to understand how they may contribute to breast cancer etiology.

Our results are based on a large sample size of women who were demographically diverse and donated healthy breast tissue. Given the dearth of research on air pollutants in relation to breast tissue characteristics, a strength of this study was the range of pollutants considered. However, it is important to note that the correlations across certain pollutants are high and independent associations may be difficult to disentangle. Each of the gaseous pollutants, NO_2_, CO, and SO_2_, is distinct criteria pollutants with varying relative contribution from different sources and is a regulatory priority of the Environmental Protection Agency, so understanding their individual health effects is important. For the PM_2.5_ components, we were able to leverage these high correlations in our *K*-means approach to identify subgroups of women who had similar patterns of exposure. This was an important consideration because PM_2.5_ is a heterogeneous mixture and studies have noted that health effects may vary by the composition of the PM_2.5_ [10, 40, 41]. Another strength of this study was the consideration of clusters of individuals based on distinct PM_2.5_ profiles that had differential associations with TDLU involution. While our clusters generally separated individuals based on high, average, or low levels of all PM_2.5_ components rather than varying combinations of the components, these findings supported our overall PM_2.5_ results.

A small breast tissue core has been shown to be generally representative of involution throughout the breast [42], but we cannot exclude the possibility of non-representative sampling of breast tissue. We adjusted for percent of fat on the slide, which is inversely related to TDLU count [5], to partially account for this possibility. A strength of this study was the inclusion of multiple measures of TDLU involution in addition to a range of different air pollutants. However, this resulted in many statistical comparisons, and thus, it is possible that some findings may be due to chance. Given this, we focused the interpretation of our results on the magnitude of point estimates, precision of confidence intervals, and trends observed in the data. Women provided their residential address at the time of tissue donation, but we did not have additional information about residential history, such as how long they had lived there or information on past residences. Therefore, we could not evaluate past air pollution exposure. Further, concentrations at a 12 × 12km grid level linked to residences do not fully account for variations in an individual’s daily activities, such as where they work, that could impact their exposure. PM_2.5_ total mass data came from the EPA’s Downscaler Model that combines monitoring data (direct ambient measurements, but sparse geographic coverage) with CMAQ data (modeled estimates at all 12 × 12km grids across the USA, but subject to calibration/accuracy of the modeling parameters) [22]. While this hinges on both sources’ strengths to increase the accuracy of the exposure assessment, non-differential exposure measurement error is still likely. Further, the components of PM_2.5_ and gaseous pollutants were available from CMAQ-modeled data alone and are less accurate than the PM_2.5_ total mass estimates from the Downscaler Model. One study reported that while fused models outperformed raw CMAQ data, the CMAQ predictions of PM_2.5_ and certain components were within recommended model performance criteria [43]. While we examined five components of PM_2.5_, information on other components of PM_2.5_ that may be carcinogenic, such as trace metals and polycyclic aromatic hydrocarbons, was not available from CMAQ. Finally, women in the Komen Tissue Bank volunteered to donate healthy breast tissue and a majority lived in Indiana, which may limit the generalizability of our results.

## Conclusions

Our results suggest outdoor air pollution exposure may affect characteristics of normal breast tissue through reduced TDLU involution as measured by TDLU counts. We specifically found that PM_2.5_ total mass, most of the individual components of PM_2.5_ that were considered, and gaseous pollutants, including those that are proxies of traffic-related air pollution, were non-linearly associated with higher TDLU counts. Future studies of lobular involution of the breast should continue to examine geographic variations in air pollution and consider other air pollution exposure metrics that we were not able to examine in this study. More research on the relationship between widespread environmental exposures and TDLU involution will enhance our understanding of the mechanisms underlying the role of the environment in breast carcinogenesis.

## Supplementary information

**Additional file 1: Supplemental Table 1**. State of residence at time of tissue donation. **Supplemental Table 2.** Mean concentrations and quartile cut-points for PM_2.5_ total mass^a^, PM_2.5_ components^b^, and gaseous pollutants^c.^ **Supplemental Table 3.** Associations between PM_2.5_ total mass^a^ and terminal duct lobular unit (TDLU) counts restricted to Indiana residents. **Supplemental Table 4.** Associations between PM_2.5_ total mass^a^ and TDLU span. **Supplemental Table 5.** Associations between PM_2.5_ total mass^a^ and acini count/TDLU. **Supplemental Table 6.** Associations between PM_2.5_ components and gaseous pollutants^a^ and terminal duct lobular unit (TDLU) counts restricted to Indiana residents. **Supplemental Table 7.** Associations between PM_2.5_ components and gaseous pollutants^a^ and TDLU span. **Supplemental Table 8.** Associations between PM_2.5_ components and gaseous pollutants^a^ and acini count/TDLU. **Supplemental Table 9.** State of residence at time of tissue donation by PM_2.5_ component cluster. **Supplemental Table 10**. Participant characteristics by PM_2.5_ component cluster. **Supplemental Figure 1**. Directed acyclic graph for the relationship between air pollution and TDLU measures. **Supplemental Figure 2**. Spearman correlations between PM_2.5_ total mass, PM_2.5_ components, and gaseous pollutants.

## Data Availability

Requests for Komen Tissue Bank data, including the data used in this manuscript, can be requested through the study website (https://komentissuebank.iu.edu/researchers/).
