# Supplementary material for: Outdoor air pollution and terminal duct lobular involution of the normal breast
Source: Breast Cancer Res. 2020 Sep 24;22:100. doi: 10.1186/s13058-020-01339-x (PMC7513536; doi:10.1186/s13058-020-01339-x)

| **Supplemental Table 1. State of residence at time of tissue donation** | | | | | | |
| --- | --- | --- | --- | --- | --- | --- |
|  | **Overall** | | **Premenopausal** | | **Postmenopausal** | |
| **State** | **N** | **%** | **N** | **%** | **N** | **%** |
| Alabama | 2 | 0.1 | 1 | 0.1 | 1 | 0.2 |
| Arizona | 2 | 0.1 | 1 | 0.1 | 1 | 0.2 |
| California | 4 | 0.2 | 3 | 0.2 | 1 | 0.2 |
| Connecticut | 1 | 0.1 | 1 | 0.1 | 0 | - |
| Washington, DC | 1 | 0.1 | 1 | 0.1 | 0 | - |
| Florida | 10 | 0.5 | 7 | 0.5 | 3 | 0.5 |
| Georgia | 2 | 0.1 | 1 | 0.1 | 1 | 0.2 |
| Illinois | 23 | 1.2 | 23 | 1.7 | 0 | - |
| Indiana | 1,687 | 88.6 | 1,162 | 87.9 | 517 | 90.1 |
| Iowa | 1 | 0.1 | 1 | 0.1 | 0 | - |
| Kansas | 3 | 0.2 | 3 | 0.2 | 0 | - |
| Kentucky | 84 | 4.4 | 49 | 3.7 | 35 | 6.1 |
| Maryland | 2 | 0.1 | 2 | 0.2 | 0 | - |
| Michigan | 9 | 0.5 | 5 | 0.4 | 4 | 0.2 |
| Minnesota | 2 | 0.1 | 2 | 0.2 | 0 | - |
| Missouri | 1 | 0.1 | 1 | 0.1 | 0 | - |
| North Carolina | 4 | 0.2 | 4 | 0.3 | 0 | - |
| New York | 1 | 0.1 | 0 | - | 1 | 0.2 |
| New Jersey | 2 | 0.1 | 1 | 0.1 | 1 | 0.2 |
| Ohio | 47 | 2.5 | 44 | 3.3 | 3 | 0.5 |
| Pennsylvania | 3 | 0.2 | 2 | 0.2 | 1 | 0.2 |
| Tennessee | 2 | 0.1 | 2 | 0.2 | 0 | - |
| Texas | 4 | 0.2 | 1 | 0.1 | 3 | 0.5 |
| Utah | 1 | 0.1 | 0 | - | 1 | 0.2 |
| Vermont | 1 | 0.1 | 0 | - | 1 | 0.2 |
| Virginia | 1 | 0.1 | 1 | 0.1 | 0 | - |
| Wisconsin | 4 | 0.2 | 4 | 0.3 | 0 | - |

Note: Percentages may not sum to 100% due to rounding

| **Supplemental Table 2. Mean concentrations and quartile cut-points for PM_2.5_ total mass^a^, PM_2.5_ components^b^, and gaseous pollutants^c^** | | | | | | | |  |
| --- | --- | --- | --- | --- | --- | --- | --- | --- |
|  | **Mean** | **SD** | **Min** | **p25** | **p50** | **p75** | **Max** | |
| **PM_2.5_ total mass (µg/m^3^)** | 12.5 | 0.7 | 6.2 | 12.3 | 12.7 | 12.8 | 14.0 | |
|  |  |  |  |  |  |  |  | |
| **PM_2.5_ components (µg/m^3^)** |  |  |  |  |  |  |  | |
| **SO_4_** | 2.4 | 0.2 | 0.5 | 2.4 | 2.4 | 2.5 | 3.0 | |
| **NO_3_** | 1.9 | 0.3 | 0.1 | 1.8 | 2.0 | 2.1 | 2.8 | |
| **NH_4_** | 1.3 | 0.1 | 0.1 | 1.2 | 1.3 | 1.4 | 1.6 | |
| **EC** | 1.3 | 0.5 | 0.2 | 0.9 | 1.3 | 1.6 | 3.0 | |
| **OC** | 2.0 | 0.5 | 0.7 | 1.5 | 2.0 | 2.4 | 5.0 | |
|  |  |  |  |  |  |  |  | |
| **Gaseous pollutants (ppb)** |  |  |  |  |  |  |  | |
| **CO** | 223.4 | 52.6 | 120.5 | 180.0 | 229.3 | 265.7 | 440.8 | |
| **NO** | 4.6 | 3.3 | 0.1 | 1.6 | 3.8 | 6.6 | 25.8 | |
| **NO_2_** | 13.1 | 4.8 | 1.6 | 9.3 | 13.4 | 16.3 | 29.2 | |
| **SO_2_** | 2.4 | 0.8 | 0.3 | 1.8 | 2.3 | 2.7 | 5.2 | |
| ^a^ PM_2.5_ total mass concentrations from a Downscaler model of fused monitoring and CMAQ data  ^b^ PM_2.5_ component (SO_4_, NO_3_, NH_4_, EC, OC) concentrations from “raw” CMAQ data  ^c^ Gaseous pollutants (CO, NO, NO_2_, SO_2_) concentrations from “raw” CMAQ data | | | | | | | |  |

| **Supplemental Table 3. Associations between PM_2.5_ total mass^a^ and terminal duct lobular unit (TDLU) counts restricted to Indiana residents** | | | | | | |
| --- | --- | --- | --- | --- | --- | --- |
|  | **All women** | | **Premenopausal** | | **Postmenopausal** | |
|  | **N** | **RR^b,c^ (95% CI)** | **N** | **RR^b,c^ (95% CI)** | **N** | **RR^b,c^ (95% CI)** |
| **PM_2.5_ total mass (µg/m^3^)** |  |  |  |  |  |  |
| Quartile 1 | 325 | Ref. | 217 | Ref. | 107 | Ref. |
| Quartile 2 | 429 | 1.24 (1.04, 1.49) | 313 | 1.33 (1.08, 1.64) | 111 | 0.97 (0.68, 1.39) |
| Quartile 3 | 513 | 1.19 (0.99, 1.42) | 327 | 1.28 (1.04, 1.59) | 184 | 0.99 (0.71, 1.37) |
| Quartile 4 | 420 | 1.12 (0.93, 1.35) | 305 | 1.12 (0.91, 1.39) | 115 | 1.18 (0.83, 1.67) |
| IQR increase^d^ | 1,687 | 1.06 (0.96, 1.16) | 1,162 | 1.07 (0.95, 1.20) | 517 | 1.05 (0.89, 1.24) |
| ^a^ PM_2.5_ total mass concentrations (µg/m^3^) were estimated by the EPA’s Downscaler model of fused CMAQ and monitoring data  ^b^ Adjusted for age, smoking status, education, race/ethnicity, BMI, and percent of fat on the slide  ^c^ From a zero-inflated negative binomial regression model. Zero model based on same covariates as main model  ^d^ IQR increase= 0.5 µg/m^3^ | | | | | | |

| **Supplemental Table 4. Associations between PM_2.5_ total mass^a^ and TDLU span** | | | | | | |
| --- | --- | --- | --- | --- | --- | --- |
|  | **All women** | | **Premenopausal** | | **Postmenopausal** | |
|  | **N^b^** | **OR^c,d^ (95% CI)** | **N** | **OR^c,d^ (95% CI)** | **N** | **OR^c,d^ (95% CI)** |
| **PM_2.5_ total mass (µg/m^3^)** |  |  |  |  |  |  |
| Quartile 1 | 306 | Ref. | 234 | Ref. | 71 | Ref. |
| Quartile 2 | 298 | 0.94 (0.69, 1.28) | 229 | 1.01 (0.72, 1.44) | 66 | 0.70 (0.36, 1.35) |
| Quartile 3 | 350 | 0.74 (0.54, 1.01) | 232 | 0.69 (0.49, 0.99) | 116 | 0.83 (0.46, 1.49) |
| Quartile 4 | 308 | 0.75 (0.55, 1.03) | 231 | 0.73 (0.51, 1.04) | 77 | 0.82 (0.44, 1.53) |
| IQR increase^e^ | 1,262 | 0.99 (0.90, 1.08) | 926 | 0.99 (0.90, 1.09) | 330 | 0.98 (0.79, 1.20) |
| ^a^ PM_2.5_ total mass concentrations (µg/m^3^) were estimated by the EPA’s Downscaler model of fused CMAQ and monitoring data  ^b^ 644 H&E images had 0 TDLU count and therefore no data for TDLU span  ^c^ Adjusted for age, smoking status, education, race/ethnicity, BMI, and percent of fat on the slide  ^d^ From an ordinal logistic regression model. Outcome TDLU span was categorized based on tertiles (53-266, >266-382, >382)  ^e^ IQR increase= 0.5 µg/m^3^ | | | | | | |

| **Supplemental Table 5. Associations between PM_2.5_ total mass^a^ and acini count/TDLU** | | | | | | |
| --- | --- | --- | --- | --- | --- | --- |
|  | **All women** | | **Premenopausal** | | **Postmenopausal** | |
|  | **N^b^** | **OR^c,d^  (95% CI)** | **N** | **OR^c,d^ (95% CI)** | **N** | **OR^c,d^ (95% CI)** |
| **PM_2.5_ total mass (µg/m^3^)** |  |  |  |  |  |  |
| Quartile 1 | 306 | Ref. | 234 | Ref. | 71 | Ref. |
| Quartile 2 | 298 | 1.05 (0.77, 1.50) | 229 | 1.12 (0.79, 1.59) | 66 | 0.59 (0.26, 1.32) |
| Quartile 3 | 350 | 0.85 (0.61, 1.18) | 232 | 0.89 (0.62, 1.28) | 116 | 0.65 (0.32, 1.32) |
| Quartile 4 | 308 | 0.97 (0.70, 1.34) | 231 | 0.97 (0.68, 1.39) | 77 | 0.94 (0.45, 2.00) |
| IQR increase^e^ | 1,262 | 1.06 (0.96, 1.17) | 926 | 1.07 (0.97, 1.19) | 330 | 1.01 (0.78, 1.32) |
| ^a^ PM_2.5_ total mass concentrations (µg/m^3^) were estimated by the EPA’s Downscaler model of fused CMAQ and monitoring data  ^b^ 644 H&E images had 0 TDLU count and therefore no data for acini count/TDLU  ^c^ Adjusted for age, smoking status, education, race/ethnicity, BMI, and percent of fat on the slide  ^d^ From an ordinal logistic regression model. Outcome acini/TDLU was categorized based on tertiles (1, >1-2, >2)  ^e^ IQR increase= 0.5 µg/m^3^ | | | | | | |

| **Supplemental Table 6. Associations between PM_2.5_ components and gaseous pollutants^a^ and terminal duct lobular unit (TDLU) counts restricted to Indiana residents** | | | | | | |
| --- | --- | --- | --- | --- | --- | --- |
|  | **All women** | | **Premenopausal** | | **Postmenopausal** | |
|  | **N** | **RR^b,c^ (95% CI)** | **N** | **RR^b,c^ (95% CI)** | **N** | **RR^b,c^ (95% CI)** |
| **PM_2.5_ components (µg/m^3^)** | | | | | | |
| **SO_4_** |  |  |  |  |  |  |
| Quartile 1 | 358 | Ref. | 256 | Ref. | 100 | Ref. |
| Quartile 2 | 494 | 1.13 (0.96, 1.34) | 338 | 1.15 (0.95, 1.40) | 155 | 1.11 (0.80, 1.54) |
| Quartile 3 | 449 | 0.94 (0.79, 1.11) | 293 | 0.94 (0.76, 1.15) | 154 | 0.98 (0.71, 1.36) |
| Quartile 4 | 386 | 1.02 (0.86, 1.22) | 275 | 1.04 (0.85, 1.27) | 108 | 1.05 (0.73, 1.51) |
| IQR increase^d^ | 1,687 | 1.00 (0.95, 1.06) | 1,162 | 1.00 (0.94, 1.07) | 517 | 1.02 (0.91, 1.14) |
| **NO_3_** |  |  |  |  |  |  |
| Quartile 1 | 314 | Ref. | 208 | Ref. | 102 | Ref. |
| Quartile 2 | 426 | 1.03 (0.85, 1.23) | 307 | 1.02 (0.82, 1.26) | 119 | 1.02 (0.71, 1.47) |
| Quartile 3 | 504 | 1.09 (0.91, 1.31) | 347 | 1.08 (0.88, 1.33) | 154 | 1.10 (0.78, 1.56) |
| Quartile 4 | 443 | 1.14 (0.95, 1.37) | 300 | 1.14 (0.92, 1.42) | 142 | 1.13 (0.79, 1.60) |
| IQR increase^d^ | 1,687 | 1.04 (0.97, 1.11) | 1,162 | 1.04 (0.96, 1.13) | 517 | 1.03 (0.90, 1.17) |
| **NH_4_** |  |  |  |  |  |  |
| Quartile 1 | 329 | Ref. | 210 | Ref. | 115 | Ref. |
| Quartile 2 | 438 | 1.09 (0.91, 1.31) | 318 | 1.04 (0.84, 1.28) | 119 | 1.20 (0.84, 1.70) |
| Quartile 3 | 419 | 0.98 (0.82, 1.18) | 268 | 0.97 (0.78, 1.21) | 148 | 1.02 (0.73, 1.42) |
| Quartile 4 | 501 | 1.07 (0.90, 1.28) | 366 | 1.02 (0.83, 1.25) | 135 | 1.28 (0.91, 1.79) |
| IQR increase^d^ | 1,687 | 1.02 (0.93, 1.12) | 1,162 | 1.01 (0.91, 1.13) | 517 | 1.06 (0.90, 1.25) |
| **EC** |  |  |  |  |  |  |
| Quartile 1 | 365 | Ref. | 264 | Ref. | 100 | Ref. |
| Quartile 2 | 458 | 1.34 (1.13, 1.59) | 322 | 1.44 (1.18, 1.75) | 131 | 1.06 (0.75, 1.50) |
| Quartile 3 | 375 | 1.10 (0.91, 1.33) | 242 | 1.16 (0.93, 1.45) | 131 | 0.98 (0.69, 1.38) |
| Quartile 4 | 489 | 1.17 (0.99, 1.40) | 334 | 1.20 (0.98, 1.47) | 155 | 1.13 (0.81, 1.59) |
| IQR increase^d^ | 1,687 | 1.05 (0.95, 1.16) | 1,162 | 1.06 (0.94, 1.19) | 517 | 1.06 (0.87, 1.28) |
| **OC** |  |  |  |  |  |  |
| Quartile 1 | 412 | Ref. | 294 | Ref. | 116 | Ref. |
| Quartile 2 | 442 | 1.19 (1.00, 1.41) | 314 | 1.23 (1.02, 1.50) | 126 | 1.06 (0.75, 1.48) |
| Quartile 3 | 374 | 1.19 (1.00, 1.41) | 241 | 1.28 (1.05, 1.58) | 131 | 0.96 (0.69, 1.34) |
| Quartile 4 | 459 | 1.08 (0.91, 1.28) | 313 | 1.11 (0.92, 1.35) | 144 | 0.99 (0.72, 1.37) |
| IQR increase^d^ | 1,687 | 1.03 (0.92, 1.15) | 1,162 | 1.04 (0.92, 1.18) | 517 | 1.03 (0.84, 1.27) |
| **Gaseous pollutants (ppb)** | | | | | | |
| **CO** |  |  |  |  |  |  |
| Quartile 1 | 391 | Ref. | 282 | Ref. | 108 | Ref. |
| Quartile 2 | 451 | 1.26 (1.06, 1.50) | 306 | 1.36 (1.12, 1.66) | 141 | 0.98 (0.69, 1.38) |
| Quartile 3 | 424 | 1.17 (0.99, 1.39) | 299 | 1.26 (1.04, 1.54) | 124 | 0.93 (0.66, 1.30) |
| Quartile 4 | 421 | 1.15 (0.96, 1.37) | 275 | 1.17 (0.95, 1.44) | 144 | 1.07 (0.77, 1.49) |
| IQR increase^d^ | 1,687 | 1.04 (0.93, 1.15) | 1,162 | 1.04 (0.93, 1.18) | 517 | 1.04 (0.86, 1.27) |
| **NO** |  |  |  |  |  |  |
| Quartile 1 | 379 | Ref. | 273 | Ref. | 103 | Ref. |
| Quartile 2 | 423 | 1.31 (1.11, 1.56) | 299 | 1.39 (1.14, 1.69) | 121 | 1.06 (0.74, 1.51) |
| Quartile 3 | 396 | 1.10 (0.91, 1.32) | 256 | 1.16 (0.94, 1.44) | 138 | 0.96 (0.69, 1.34) |
| Quartile 4 | 489 | 1.15 (0.97, 1.37) | 334 | 1.18 (0.96, 1.43) | 155 | 1.12 (0.80, 1.56) |
| IQR increase^d^ | 1,687 | 1.04 (0.94, 1.14) | 1,162 | 1.04 (0.93, 1.16) | 517 | 1.06 (0.89, 1.27) |
| **NO_2_** |  |  |  |  |  |  |
| Quartile 1 | 376 | Ref. | 272 | Ref. | 103 | Ref. |
| Quartile 2 | 417 | 1.34 (1.12, 1.59) | 292 | 1.45 (1.19, 1.78) | 121 | 1.00 (0.70, 1.42) |
| Quartile 3 | 470 | 1.11 (0.93, 1.32) | 312 | 1.18 (0.96, 1.44) | 155 | 0.96 (0.69, 1.33) |
| Quartile 4 | 424 | 1.18 (0.99, 1.42) | 286 | 1.24 (1.01, 1.52) | 138 | 1.08 (0.77, 1.51) |
| IQR increase^d^ | 1,687 | 1.04 (0.95, 1.15) | 1,162 | 1.05 (0.94, 1.17) | 517 | 1.04 (0.88, 1.24) |
| **SO_2_** |  |  |  |  |  |  |
| Quartile 1 | 376 | Ref. | 256 | Ref. | 119 | Ref. |
| Quartile 2 | 371 | 1.16 (0.97, 1.39) | 265 | 1.20 (0.97, 1.48) | 104 | 0.99 (0.70, 1.41) |
| Quartile 3 | 502 | 1.00 (0.85, 1.18) | 333 | 0.98 (0.80, 1.19) | 167 | 1.08 (0.79, 1.47) |
| Quartile 4 | 438 | 0.99 (0.83, 1.19) | 308 | 1.02 (0.83, 1.25) | 127 | 0.92 (0.66, 1.28) |
| IQR increase^d^ | 1,687 | 0.98 (0.92, 1.05) | 1,162 | 0.98 (0.91, 1.06) | 517 | 1.00 (0.88, 1.15) |
| ^a^ PM_2.5_ component (µg/m^3^) and gaseous pollutant concentrations (ppb) were estimated from “raw” CMAQ data  ^b^ Adjusted for age, smoking status, education, race/ethnicity, BMI, and percent of fat on the slide  ^c^ From a zero-inflated negative binomial regression model. Zero model based on same covariates as main model  ^d^ IQR increase: SO_4_=0.1 µg/m^3^, NO_3_=0.3 µg/m^3^, NH_4_=0.1 µg/m^3^, EC=0.8 µg/m^3^, OC=0.8 µg/m^3^, CO=85.6 ppb, NO=5.0 ppb, NO_2_= 7.1 ppb, SO_2_= 0.8 ppb | | | | | | |

| **Supplemental Table 7. Associations between PM_2.5_ components and gaseous pollutants ^a^ and TDLU span** | | | | | | |
| --- | --- | --- | --- | --- | --- | --- |
|  | **All women** | | **Premenopausal** | | **Postmenopausal** | |
|  | **N^b^** | **OR^c,d^ (95% CI)** | **N** | **OR^c,d^ (95% CI)** | **N** | **OR^c,d^ (95% CI)** |
| **PM_2.5_ components (µg/m^3^)** | | | | | | |
| **SO_4_** |  |  |  |  |  |  |
| Quartile 1 | 291 | Ref. | 223 | Ref. | 67 | Ref. |
| Quartile 2 | 352 | 0.96 (0.71, 1.30) | 250 | 0.88 (0.62, 1.24) | 101 | 1.35 (0.73, 2.51) |
| Quartile 3 | 316 | 0.98 (0.72, 1.34) | 219 | 0.88 (0.61, 1.25) | 95 | 1.41 (0.76, 2.63) |
| Quartile 4 | 303 | 0.91 (0.66, 1.24) | 234 | 0.81 (0.57, 1.15) | 67 | 1.37 (0.70, 2.70) |
| IQR increase^e^ | 1,262 | 1.01 (0.94, 1.08) | 926 | 0.99 (0.91, 1.08) | 330 | 1.05 (0.90, 1.21) |
| **NO_3_** |  |  |  |  |  |  |
| Quartile 1 | 289 | Ref. | 223 | Ref. | 63 | Ref. |
| Quartile 2 | 300 | 0.72 (0.53, 0.98) | 227 | 0.75 (0.53, 1.07) | 73 | 0.61 (0.32, 1.16) |
| Quartile 3 | 352 | 0.77 (0.57, 1.05) | 247 | 0.88 (0.62, 1.24) | 102 | 0.51 (0.28, 0.95) |
| Quartile 4 | 321 | 0.71 (0.52, 0.97) | 229 | 0.74 (0.52, 1.05) | 92 | 0.60 (0.32, 1.11) |
| IQR increase^e^ | 1,262 | 0.94 (0.86, 1.04) | 926 | 0.97 (0.88, 1.08) | 330 | 0.84 (0.68, 1.03) |
| **NH_4_** |  |  |  |  |  |  |
| Quartile 1 | 284 | Ref. | 211 | Ref. | 70 | Ref. |
| Quartile 2 | 318 | 0.62 (0.46, 0.85) | 243 | 0.68 (0.48, 0.97) | 74 | 0.45 (0.24, 0.86) |
| Quartile 3 | 306 | 0.71 (0.52, 0.98) | 209 | 0.79 (0.55, 1.14) | 95 | 0.51 (0.28, 0.93) |
| Quartile 4 | 354 | 0.63 (0.47, 0.86) | 263 | 0.65 (0.46, 0.93) | 91 | 0.56 (0.31, 1.02) |
| IQR increase^e^ | 1,262 | 0.96 (0.86, 1.07) | 926 | 0.97 (0.86, 1.10) | 330 | 0.91 (0.72, 1.16) |
| **EC** |  |  |  |  |  |  |
| Quartile 1 | 303 | Ref. | 236 | Ref. | 66 | Ref. |
| Quartile 2 | 338 | 0.85 (0.63, 1.14) | 259 | 0.87 (0.62, 1.21) | 76 | 0.76 (0.41, 1.46) |
| Quartile 3 | 276 | 0.69 (0.49, 0.95) | 183 | 0.70 (0.48, 1.02) | 91 | 0.65 (0.34, 1.21) |
| Quartile 4 | 345 | 0.73 (0.54, 0.99) | 249 | 0.69 (0.49, 0.97) | 97 | 0.85 (0.46, 1.57) |
| IQR increase^e^ | 1,262 | 0.80 (0.67, 0.96) | 926 | 0.79 (0.65, 0.96) | 330 | 0.82 (0.57, 1.17) |
| **OC** |  |  |  |  |  |  |
| Quartile 1 | 307 | Ref. | 237 | Ref. | 69 | Ref. |
| Quartile 2 | 332 | 0.84 (0.62, 1.13) | 249 | 0.91 (0.65, 1.28) | 81 | 0.64 (0.34, 1.19) |
| Quartile 3 | 272 | 0.65 (0.48, 0.90) | 185 | 0.64 (0.44, 0.92) | 86 | 0.68 (0.37, 1.27) |
| Quartile 4 | 351 | 0.79 (0.59, 1.07) | 255 | 0.86 (0.61, 1.21) | 94 | 0.60 (0.33, 1.11) |
| IQR increase^e^ | 1,262 | 0.81 (0.67, 0.97) | 926 | 0.82 (0.67, 1.01) | 330 | 0.75 (0.51, 1.11) |
| **Gaseous pollutants (ppb)** | | | | | | |
| **CO** |  |  |  |  |  |  |
| Quartile 1 | 303 | Ref. | 232 | Ref. | 70 | Ref. |
| Quartile 2 | 232 | 0.89 (0.66, 1.20) | 251 | 0.84 (0.60, 1.18) | 79 | 1.05 (0.56, 1.96) |
| Quartile 3 | 306 | 0.78 (0.58, 1.07) | 219 | 0.77 (0.54, 1.10) | 86 | 0.84 (0.45, 1.56) |
| Quartile 4 | 321 | 0.81 (0.59, 1.10) | 224 | 0.80 (0.56, 1.13) | 95 | 0.80 (0.44, 1.48) |
| IQR increase^e^ | 1,262 | 0.79 (0.66, 0.95) | 926 | 0.79 (0.65, 0.97) | 330 | 0.77 (0.53, 1.11) |
| **NO** |  |  |  |  |  |  |
| Quartile 1 | 309 | Ref. | 238 | Ref. | 68 | Ref. |
| Quartile 2 | 309 | 0.90 (0.67, 1.22) | 242 | 0.94 (0.67, 1.32) | 66 | 0.78 (0.40, 1.50) |
| Quartile 3 | 294 | 0.70 (0.51, 0.97) | 193 | 0.74 (0.51, 1.07) | 99 | 0.61 (0.33, 1.13) |
| Quartile 4 | 350 | 0.76 (0.56, 1.02) | 253 | 0.72 (0.51, 1.02) | 97 | 0.83 (0.46, 1.53) |
| IQR increase^e^ | 1,262 | 0.83 (0.71, 0.98) | 926 | 0.82 (0.68, 0.98) | 330 | 0.88 (0.63, 1.24) |
| **NO_2_** |  |  |  |  |  |  |
| Quartile 1 | 303 | Ref. | 233 | Ref. | 69 | Ref. |
| Quartile 2 | 313 | 0.94 (0.69, 1.27) | 242 | 0.96 (0.68, 1.35) | 68 | 0.86 (0.45, 1.65) |
| Quartile 3 | 341 | 0.71 (0.52, 0.96) | 233 | 0.72 (0.51, 1.02) | 106 | 0.66 (0.36, 1.20) |
| Quartile 4 | 305 | 0.76 (0.56, 1.04) | 218 | 0.72 (0.50, 1.03) | 87 | 0.89 (0.47, 1.65) |
| IQR increase^e^ | 1,262 | 0.79 (0.67, 0.93) | 926 | 0.79 (0.65, 0.95) | 330 | 0.78 (0.56, 1.08) |
| **SO_2_** |  |  |  |  |  |  |
| Quartile 1 | 296 | Ref. | 217 | Ref. | 79 | Ref. |
| Quartile 2 | 273 | 0.73 (0.53, 1.00) | 204 | 0.77 (0.53, 1.10) | 67 | 0.62 (0.32, 1.17) |
| Quartile 3 | 369 | 0.77 (0.57, 1.04) | 266 | 0.73 (0.52, 1.03) | 101 | 0.90 (0.51, 1.59) |
| Quartile 4 | 324 | 0.75 (0.55, 1.02) | 239 | 0.77 (0.54, 1.10) | 83 | 0.64 (0.35, 1.18) |
| IQR increase^e^ | 1,262 | 0.94 (0.84, 1.06) | 926 | 0.95 (0.83, 1.08) | 330 | 0.91 (0.71, 1.17) |
| ^a^ PM_2.5_ component (µg/m^3^) and gaseous pollutant (ppb) concentrations from “raw” CMAQ data  ^b^ 644 H&E images had 0 TDLU count and therefore no data for TDLU span  ^c^ Adjusted for age, smoking status, education, race/ethnicity, BMI, and percent of fat on the slide  ^d^ From an ordinal logistic regression model. Outcome TDLU span was categorized based on tertiles (53-266, >266-382, >382)  ^e^ IQR increase: SO_4_=0.1 µg/m^3^, NO_3_=0.3 µg/m^3^, NH_4_=0.1 µg/m^3^, EC=0.78µg/m^3^, OC=0.8 µg/m^3^, CO=85.6 ppb, NO=5.0 ppb, NO_2_= 7.1 ppb, SO_2_= 0.8 ppb | | | | | | |

| **Supplemental Table 8. Associations between PM_2.5_ components and gaseous pollutants^a^ and acini count/TDLU** | | | | | | |
| --- | --- | --- | --- | --- | --- | --- |
|  | **All women** | | **Premenopausal** | | **Postmenopausal** | |
|  | **N^b^** | **OR^c,d^ (95% CI)** | **N** | **OR^c,d^ (95% CI)** | **N** | **OR^c,d^ (95% CI)** |
| **PM_2.5_ components (µg/m^3^)** | | | | | | |
| **SO_4_** |  |  |  |  |  |  |
| Quartile 1 | 291 | Ref. | 223 | Ref. | 67 | Ref. |
| Quartile 2 | 352 | 1.04 (0.76, 1.43) | 250 | 0.96 (0.68, 1.36) | 101 | 1.48 (0.70, 3.13) |
| Quartile 3 | 316 | 0.85 (0.61, 1.17) | 219 | 0.86 (0.60, 1.24) | 95 | 0.79 (0.35, 1.78) |
| Quartile 4 | 303 | 0.93 (0.67, 1.29) | 234 | 0.85 (0.60, 1.22) | 67 | 1.37 (0.61, 3.10) |
| IQR increase^e^ | 1,262 | 1.03 (0.96, 1.11) | 926 | 1.02 (0.94, 1.11) | 330 | 1.10 (0.90, 1.35) |
| **NO_3_** |  |  |  |  |  |  |
| Quartile 1 | 289 | Ref. | 223 | Ref. | 63 | Ref. |
| Quartile 2 | 300 | 0.95 (0.68, 1.31) | 227 | 0.99 (0.69, 1.41) | 73 | 0.73 (0.32, 1.63) |
| Quartile 3 | 352 | 1.02 (0.75, 1.40) | 247 | 1.07 (0.75, 1.51) | 102 | 0.76 (0.36, 1.60) |
| Quartile 4 | 321 | 0.88 (0.63, 1.21) | 229 | 0.84 (0.59, 1.21) | 92 | 0.98 (0.47, 2.07) |
| IQR increase^e^ | 1,262 | 1.05 (0.95, 1.15) | 926 | 1.05 (0.94, 1.17) | 330 | 1.01 (0.78, 1.30) |
| **NH_4_** |  |  |  |  |  |  |
| Quartile 1 | 284 | Ref. | 211 | Ref. | 70 | Ref. |
| Quartile 2 | 318 | 0.93 (0.67, 1.28) | 243 | 0.90 (0.63, 1.28) | 74 | 0.89 (0.40, 1.98) |
| Quartile 3 | 306 | 1.01 (0.73, 1.39) | 209 | 1.06 (0.74, 1.54) | 95 | 0.78 (0.36, 1.67) |
| Quartile 4 | 354 | 0.97 (0.71, 1.33) | 263 | 0.91 (0.64, 1.29) | 91 | 1.27 (0.62, 2.63) |
| IQR increase^e^ | 1,262 | 1.05 (0.94, 1.18) | 926 | 1.05 (0.92, 1.18) | 330 | 1.11 (0.82, 1.50) |
| **EC** |  |  |  |  |  |  |
| Quartile 1 | 303 | Ref. | 236 | Ref. | 66 | Ref. |
| Quartile 2 | 338 | 0.89 (0.66, 1.22) | 259 | 0.88 (0.63, 1.24) | 76 | 0.75 (0.34, 1.62) |
| Quartile 3 | 276 | 0.82 (0.58, 1.169) | 183 | 0.89 (0.61, 1.31) | 91 | 0.59 (0.27, 1.27) |
| Quartile 4 | 345 | 0.81 (0.59, 1.12) | 248 | 0.78 (0.55, 1.10) | 97 | 0.89 (0.43, 1.85) |
| IQR increase^e^ | 1,262 | 0.90 (0.75, 1.09) | 926 | 0.89 (0.73, 1.09) | 330 | 0.92 (0.59, 1.43) |
| **OC** |  |  |  |  |  |  |
| Quartile 1 | 307 | Ref. | 237 | Ref. | 69 | Ref. |
| Quartile 2 | 332 | 1.03 (0.75, 1.40) | 249 | 1.03 (0.73, 1.46) | 81 | 0.96 (0.45, 2.06) |
| Quartile 3 | 272 | 0.72 (0.52, 1.01) | 185 | 0.69 (0.48, 1.01) | 86 | 0.78 (0.36, 1.69) |
| Quartile 4 | 351 | 1.00 (0.73, 1.37) | 255 | 1.04 (0.74, 1.47) | 94 | 0.85 (0.40, 1.78) |
| IQR increase | 1,262 | 0.95 (0.78, 1.15) | 926 | 0.95 (0.78, 1.17) | 330 | 0.90 (0.56, 1.46) |
| **Gaseous pollutants (ppb)** | | | | | | |
| **CO** |  |  |  |  |  |  |
| Quartile 1 | 303 | Ref. | 232 | Ref. | 70 | Ref. |
| Quartile 2 | 332 | 0.91 (0.67, 1.25) | 251 | 0.90 (0.63, 1.27) | 79 | 0.81 (0.38, 1.75) |
| Quartile 3 | 306 | 0.92 (0.67, 1.28) | 219 | 0.85 (0.60, 1.22) | 86 | 1.06 (0.51, 2.19) |
| Quartile 4 | 321 | 0.86 (0.62, 1.19) | 224 | 0.90 (0.60, 1.29) | 95 | 0.62 (0.29, 1.32) |
| IQR increase^e^ | 1,262 | 0.90 (0.74, 1.08) | 926 | 0.90 (0.73, 1.10) | 330 | 0.85 (0.54, 1.33) |
| **NO** |  |  |  |  |  |  |
| Quartile 1 | 309 | Ref. | 238 | Ref. | 68 | Ref. |
| Quartile 2 | 309 | 0.88 (0.64, 1.20) | 242 | 0.91 (0.64, 1.28) | 66 | 0.57 (0.26, 1.29) |
| Quartile 3 | 294 | 0.84 (0.60, 1.18) | 193 | 0.94 (0.65, 1.37) | 99 | 0.55 (0.26, 1.15) |
| Quartile 4 | 350 | 0.81 (0.64, 1.20) | 253 | 0.79 (0.56, 1.12) | 97 | 0.81 (0.40, 1.65) |
| IQR increase^e^ | 1,262 | 0.90 (0.76, 1.07) | 926 | 0.89 (0.74, 1.08) | 330 | 0.96 (0.63, 1.45) |
| **NO_2_** |  |  |  |  |  |  |
| Quartile 1 | 303 | Ref. | 233 | Ref. | 69 | Ref. |
| Quartile 2 | 313 | 0.95 (0.69, 1.30) | 242 | 0.94 (0.67, 1.33) | 68 | 0.76 (0.34, 1.68) |
| Quartile 3 | 341 | 0.87 (0.63, 1.20) | 233 | 0.92 (0.64, 1.31) | 106 | 0.68 (0.33, 1.42) |
| Quartile 4 | 305 | 0.82 (0.59, 1.15) | 218 | 0.77 (0.54, 1.11) | 87 | 0.95 (0.45, 2.00) |
| IQR increase^e^ | 1,262 | 0.92 (0.77, 1.09) | 926 | 0.91 (0.76, 1.10) | 330 | 0.89 (0.60, 1.32) |
| **SO_2_** |  |  |  |  |  |  |
| Quartile 1 | 296 | Ref. | 217 | Ref. | 79 | Ref. |
| Quartile 2 | 273 | 0.99 (0.71, 1.37) | 204 | 1.03 (0.71, 1.48) | 67 | 0.72 (0.33, 1.56) |
| Quartile 3 | 369 | 0.92 (0.67, 1.25) | 267 | 0.95 (0.67, 1.35) | 101 | 0.78 (0.39, 1.55) |
| Quartile 4 | 324 | 1.08 (0.78, 1.50) | 239 | 1.18 (0.82, 1.69) | 83 | 0.69 (0.33, 1.43) |
| IQR increase^e^ | 1,262 | 1.10 (0.97, 1.24) | 926 | 1.10 (0.96, 1.26) | 330 | 1.07 (0.80, 1.44) |
| ^a^ PM_2.5_ component (µg/m^3^) and gaseous pollutant (ppb) concentrations estimated from “raw” CMAQ data  ^b^ 644 H&E images had 0 TDLU count and therefore no data for acini count/TDLU  ^c^ Adjusted for age, smoking status, education, race/ethnicity, BMI, and percent of fat on the slide  ^d^ From an ordinal logistic regression model. Outcome acini/TDLU was categorized based on tertiles (1, >1-2, >2)  ^e^ IQR increase: SO_4_=0.1 µg/m^3^, NO_3_=0.3 µg/m^3^, NH_4_=0.1 µg/m^3^, EC=0.8 µg/m^3^, OC=0.8 µg/m^3^, CO=85.6 ppb, NO=5.0 ppb, NO_2_= 7.1 ppb, SO_2_= 0.8 ppb | | | | | | |

| **Supplemental Table 9. State of residence at time of tissue donation by PM_2.5_ component cluster** | | | | | | |
| --- | --- | --- | --- | --- | --- | --- |
|  | **Cluster 1 (n=31)** | | **Cluster 2 (n=1,058)** | | **Cluster 3 (n=815)** | |
| **State** | **N** | **%** | **N** | **%** | **N** | **%** |
| Alabama | 2 | 6.5 | 0 | - | 0 | - |
| Arizona | 2 | 6.5 | 0 | - | 0 | - |
| California | 3 | 9.7 | 0 | - | 1 | 0.1 |
| Connecticut | 0 | - | 1 | 0.1 | 0 | - |
| Washington, DC | 0 | - | 1 | 0.1 | 0 | - |
| Florida | 10 | 32.3 | 0 | - | 0 | - |
| Georgia | 1 | 3.2 | 1 | 0.1 | 0 | - |
| Illinois | 0 | - | 8 | 0.8 | 15 | 1.8 |
| Indiana | 0 | - | 914 | 86.4 | 773 | 94.9 |
| Iowa | 0 | - | 1 | 0.1 | 0 | - |
| Kansas | 3 | 9.7 | 0 | - | 0 | - |
| Kentucky | 0 | - | 73 | 6.9 | 11 | 1.4 |
| Maryland | 0 | - | 0 | - | 2 | 0.3 |
| Michigan | 1 | 3.2 | 7 | 0.7 | 1 | 0.1 |
| Minnesota | 1 | 3.2 | 1 | 0.1 | 0 | - |
| Missouri | 0 | - | 0 | - | 1 | 0.1 |
| North Carolina | 0 | - | 4 | 0.4 | 0 | - |
| New York | 1 | 3.2 | 0 | - | 0 | - |
| New Jersey | 0 | - | 2 | 0.2 | 0 | - |
| Ohio | 0 | - | 38 | 3.6 | 9 | 1.1 |
| Pennsylvania | 0 | - | 1 | 0.1 | 2 | 0.3 |
| Tennessee | 1 | 3.2 | 1 | 0.1 | 0 | - |
| Texas | 4 | 12.9 | 0 | - | 0 | - |
| Utah | 1 | 3.2 | 0 | - | 0 | - |
| Vermont | 1 | 3.2 | 0 | - | 0 | - |
| Virginia | 0 | - | 1 | 0.1 | 0 | - |
| Wisconsin | 0 | - | 4 | 0.4 | 0 | - |

Note: Percentages may not sum to 100% due to rounding

| **Supplemental Table 10. Participant characteristics by PM_2.5_ component cluster** | | | | | | | | |
| --- | --- | --- | --- | --- | --- | --- | --- | --- |
|  | **Overall population (n=1,904)** | | **Cluster 1**  **(n=31)** | | **Cluster 2**  **(n=1,058)** | | **Cluster 3**  **(n=815)** | |
|  | **N** | **%** | **N** | **%** | **N** | **%** | **N** | **%** |
| **Race/Ethnicity** |  |  |  |  |  |  |  |  |
| Non-Hispanic white | 1371 | 72 | 22 | 71 | 851 | 80 | 497 | 61 |
| Non-Hispanic Black | 355 | 19 | 5 | 16 | 110 | 10 | 240 | 29 |
| Asian | 29 | 2 | 0 | 6 | 16 | 2 | 13 | 2 |
| Hispanic | 136 | 7 | 2 | 6 | 74 | 7 | 58 | 7 |
| Other | 16 | 1 | 2 | 2 | 7 | 1 | 7 | 1 |
| **Highest level of education** |  |  |  |  |  |  |  |  |
| Less than high school or high school graduate | 383 | 20 | 4 | 13 | 252 | 24 | 127 | 16 |
| Vocational or technical school or associate’s | 289 | 15 | 5 | 16 | 166 | 16 | 117 | 14 |
| College degree | 655 | 34 | 6 | 19 | 358 | 34 | 289 | 35 |
| Graduate or professional degree | 456 | 24 | 13 | 42 | 211 | 20 | 232 | 28 |
| Other | 124 | 7 | 3 | 10 | 71 | 7 | 50 | 6 |
| **Cigarette smoking status** |  |  |  |  |  |  |  |  |
| Never | 1390 | 73 | 17 | 55 | 773 | 73 | 598 | 73 |
| Past | 389 | 20 | 12 | 39 | 206 | 19 | 170 | 21 |
| Current | 128 | 7 | 2 | 6 | 79 | 7 | 47 | 6 |
| **BMI** |  |  |  |  |  |  |  |  |
| <25.0 | 666 | 35 | 17 | 55 | 356 | 34 | 293 | 36 |
| 25.0-<30.0 | 531 | 28 | 3 | 10 | 298 | 28 | 229 | 28 |
| ≥30.0 | 710 | 37 | 11 | 35 | 404 | 38 | 293 | 36 |
| **Age at donation (mean, sd)** | 41.5 | 13.8 | 48.1 | 11.3 | 40.5 | 13.8 | 42.7 | 13.6 |
| **PM_2.5_ components (mean, sd)** |  |  |  |  |  |  |  |  |
| SO_4_ | 2.4 | 0.2 | 1.6 | 0.4 | 2.4 | 0.2 | 2.6 | 0.1 |
| NO_3_ | 1.9 | 0.3 | 0.7 | 0.5 | 1.8 | 0.3 | 2.1 | 0.1 |
| NH_4_ | 1.3 | 0.1 | 0.6 | 0.2 | 1.2 | 0.1 | 1.4 | 0.1 |
| EC | 1.3 | 0.5 | 0.9 | 0.6 | 0.9 | 0.3 | 1.7 | 0.2 |
| OC | 2.0 | 0.5 | 1.7 | 0.7 | 1.6 | 0.3 | 2.4 | 0.3 |

Note: Percentages may not sum to 100% due to rounding


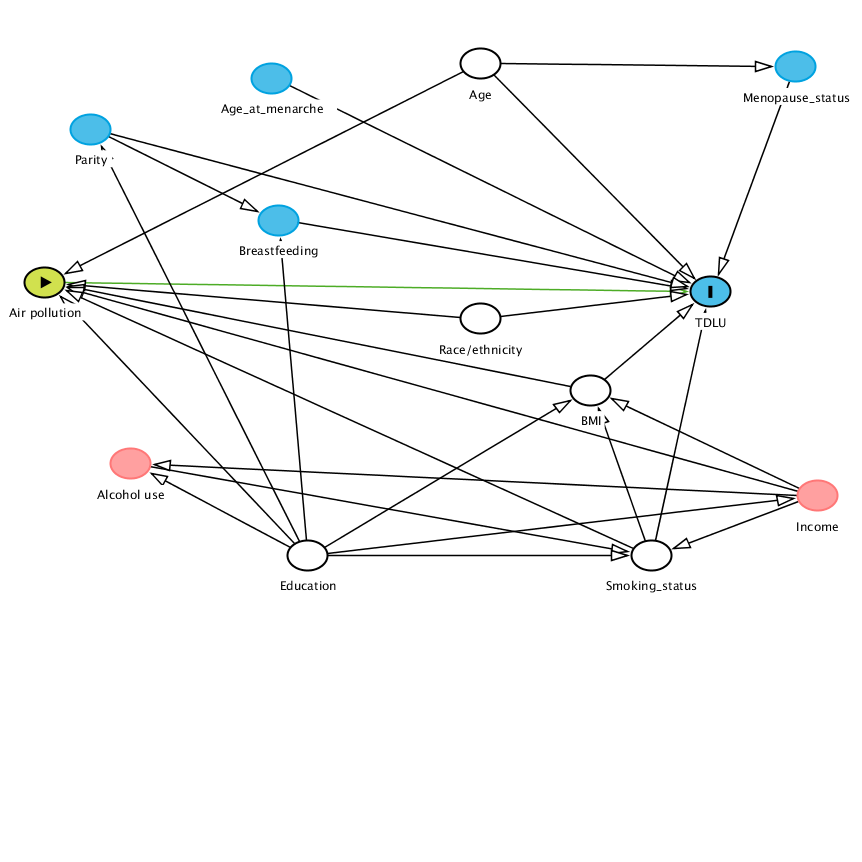
**Supplemental Figure**  **1. Directed acyclic graph for the relationship between air pollution and TDLU measures**

Description of circle and arrow colors. Green circle: exposure; blue circle with black outline: outcome; unshaded circles: variables adjusted for in the model; red circles: variables related to both the exposure and outcome (directly or indirectly) and not adjusted for in models; blue circles: variables related only to the outcome and not adjusted for in the models; green arrow: exposure-outcome path of interest; black arrows: paths accounted for in the adjusted models

**Supplemental Figure 2. Spearman correlations between PM_2.5_ total mass, PM_2.5_ components, and gaseous pollutants**


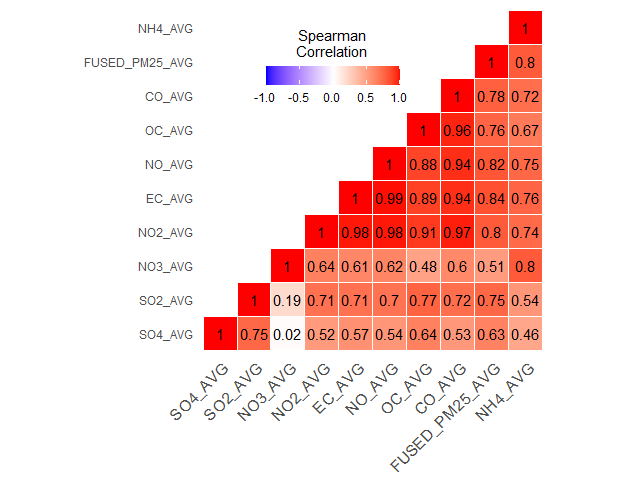

Supplement: Supplementary file 1 — Additional file 1: Supplemental Table 1. State of residence at time of tissue donation. Supplemental Table 2. Mean concentrations and quartile cut-points for PM2.5 total massa, PM2.5 componentsb, and gaseous pollutantsc. Supplemental Table 3. Associations between PM2.5 total massa and terminal duct lobular unit (TDLU) counts restricted to Indiana residents. Supplemental Table 4. Associations between PM2.5 total massa and TDLU span. Supplemental Table 5. Associations between PM2.5 total massa and acini count/TDLU. Supplemental Table 6. Associations between PM2.5 components and gaseous pollutantsa and terminal duct lobular unit (TDLU) counts restricted to Indiana residents. Supplemental Table 7. Associations between PM2.5 components and gaseous pollutantsa and TDLU span. Supplemental Table 8. Associations between PM2.5 components and gaseous pollutantsa and acini count/TDLU. Supplemental Table 9. State of residence at time of tissue donation by PM2.5 component cluster. Supplemental Table 10. Participant characteristics by PM2.5 component cluster. Supplemental Figure 1. Directed acyclic graph for the relationship between air pollution and TDLU measures. Supplemental Figure 2. Spearman correlations between PM2.5 total mass, PM2.5 components, and gaseous pollutants. [file 13058_2020_1339_MOESM1_ESM.docx]
